# Comparison of Improvement in 2-Year Survival Rate of Patients with Stage II-III Non-Small Cell Lung Cancer Treated with Different Durations of Chinese Patent Medicine: A Retrospective Cohort Study

**DOI:** 10.3389/fphar.2021.719802

**Published:** 2021-09-02

**Authors:** Li Wang, Kegang Jia, Fang Li, Chenxu Zhang, Gang Feng, Jun Du

**Affiliations:** ^1^ Oncology Department of Jiangsu Institute of Cancer Research (Jiangsu Cancer Hospital), Nanjing, China; ^2^ Department of Thoracic Surgery, Sichuan Provincial People’s Hospital, Chengdu, China; ^3^ Chengdu Diao Pharmaceutical Group Co, Ltd, Chengdu, China

**Keywords:** non-small cell lung cancer, Chinese patent medicine, Huisheng Oral Liquid, tumor stage, hypercoagulability, 2-year survival rate

## Abstract

**Background:** Chinese patent medicine is widely used among patients with malignant tumors, and current studies have shown that long-term treatment with Chinese patent medicine is related to improved outcomes of patients. Huisheng Oral Liquid is a kind of Chinese patent medicine with the effects of curing dispersion-thirst and dissipating blood stasis. However, little is known about how it affects the survival rate of patients. Thus, patients with stage II-III NSCLC (non-small-cell lung cancer) were chosen to participate in a retrospective cohort study, which was conducted to preliminarily investigate the effects of using Chinese patent medicine and Huisheng Oral Liquid for different treatment durations on patients’ 2-year survival rate and explore the prognostic factors affecting the 2-year survival rate of those patients.

**Purpose:** This work compares the effect of different durations of treatment with Chinese patent medicine and Huisheng Oral Liquid on the 2-year survival rate of patients with stage II-III NSCLC and explores the prognostic factors of the patients' 2-year survival rate.

**Methods:** This retrospective cohort study included patients with non-small cell lung cancer stage II-III according to the 2015 NCCN Guidelines: Non-Small Cell Lung Cancer. The Kaplan–Meier method was used to compare the 2-year survival rate of patients treated with different durations of Chinese medicine and Huisheng Oral Liquid. The relationship between different treatment durations and degree of improvement of 2-year survival rate was explored using the Cochran–Armitage trend test. The Cox proportional-hazards regression models were used to explore factors affecting the 2-year survival rate of patients.

**Results:** A total of 614 patients with stage II-III NSCLC diagnosed from January 2015 to December 2018 were included in this study. Patients treated with Chinese patent medicine were divided into three groups by treatment durations: < 3 months, ≥ 3 months, and ≥6 months, and those treated with Huisheng Oral Liquid were divided into < 3 months and ≥3 and ≥6 months. The results showed that ① the 2-year survival rate of patients treated with Chinese patent medicine for ≥3 months and ≥6 months was higher than that of patients treated for <3 months and the difference was statistically significant (*p* < 0.05). Further analysis of Huisheng Oral Liquid treatment revealed that ② the 2-year survival rate of patients treated with Huisheng Oral Liquid for ≥3 months was higher than that of patients treated for <3 months (*p* < 0.05). Because the total number of patients treated with Huisheng Oral Liquid for ≥6 months and the number of patients with improved outcomes were too small, there was no statistically significant difference in the 2-year survival rate between the two groups (*p* > 0.05). The results of the Cochran–Armitage trend test showed that the 2-year survival rate tended to increase with the duration of Huisheng Oral Liquid treatment (*p* < 0.05). ③ The Cox proportional -hazards regression model revealed that among all 614 patients, surgery [HR = 0.48, 95% CI = (0.34, 0.68)], chemotherapy [HR = 0.46, 95% CI = (0.31,0.67)], and treatment with Huisheng Oral Liquid for ≥3 months were protective factors [HR = 0.48, 95%CI = (0.27,0.88)], whereas male gender [HR = 1.59, 95% CI = (1.01, 2.50)] and FIB ≥4 g/L [HR = 1.95, 95% CI = (1.37, 2.77)] were risk factors.

**Conclusion:** Chinese patent medicine treatment for ≥3 months showed an improvement in the 2-year survival rate of patients with stage II-III NSCLC. Patients treated with Huisheng Oral liquid for ≥3 months also showed an improvement in the 2-year survival rate, and the 2-year survival rate tended to increase as the treatment duration increased. Finally, male and FIB ≥ 4 g/L were risk factors for prognosis.

## Introduction

According to the GLOBOCAN 2018 data (Bray et al., 2018), in 2018, approximately 2.08 million (11.6%) of the 18 million patients with new malignancies worldwide were patients with new lung cancer, and approximately 1.77 million of 9.6 million cancer deaths (18.4%) were due to the lung cancer, making it the leading cause of cancer incidence and death. Of these, more than 85% of patients are diagnosed with non-small cell lung cancer (NSCLC) (Molina et al., 2008), but the 5-year expected survival rate of these NSCLC patients is only about 15% (Chen et al., 2014a; Siegel et al., 2018), due to frequent tumor recurrence and metastasis (Ko et al., 2018). Moreover, factors such as tumor neovascularization, blood hypercoagulation, and tumor microenvironment accompanying recurrence and metastasis (Goudar and Vlahovic, 2008; Marinho and Takagaki, 2008; Altorki et al., 2019; Mascaux et al., 2019) in turn promote tumor recurrence and metastasis (Popper, 2016). Studies have shown that hypercoagulability was closely related to platelets and fibrinogen and promoted the spread of tumors (Palumbo et al., 2005). Nakano K et al. (Nakano et al., 2018) have considered that high PLT (platelet count), FIB (fibrinogen), and D-Dimer (D-Dimer) were associated with poor prognosis in patients with malignant tumors. Although the continuous development of targeted therapy and immunotherapy in recent years has significantly improved the 5-year survival rate of patients with non-small cell lung cancer and the 5-year survival rate of patients with TPS ≥50% after immunotherapy could reach 31.9% (Reck et al., 2019), high treatment costs and adverse reactions also deterred many patients from adhering to those therapies. Chinese patent medicine (CPM), as one of the development products under the theory of traditional Chinese medicine, is composed of natural ingredients and has been proved to be safe and effective (Huang et al., 2020) in enhancing the efficacy of conventional treatments, alleviating adverse reactions (Chen et al., 2014b) and improving the patients’ quality of life (Su et al., 2020). CPM is also more cost-effective to most people than western medicine and has been widely used in China. Different from chemotherapy, targeted therapy, and immunotherapy, the efficacy of CPM is mild (Qi et al., 2015); however, long-term treatment duration can help achieve good outcomes (Chen et al., 2014b). Researchers have found that the use of CPM for more than 6 months is a protective factor against recurrence and metastasis of colon cancer (Liao et al., 2017), and one investigator has found that long-term use of CPM is an independent protective factor affecting the prognosis of patients with NSCLC (Chen et al., 2014b). Huisheng Oral Liquid (HSOL), a type of CPM, has been widely used in patients with primary liver cancer and lung cancer for the 21 years since it has been on the market. Available animal studies have shown that it could reduce the incidence of lung metastasis of tumors (Wang et al., 2014) and decrease the PLT and FIB level in SD rats (Liu et al., 2013). The results of an additional animal study also showed that it could reduce FIB level, downregulate the expression of related factors in C57 mice, and reduce blood hypercoagulation, thereby helping inhibit malignant tumor development (Chen et al., 2019). However, studies investigating the relationship between different treatment durations of HSOL and the respective outcomes are rare. Under this background, we conducted a retrospective cohort study to explore the abovementioned relationship and study the potential factors affecting the prognosis of patients.

## Methods

### Patients Data

After obtaining approval from the hospitals’ ethics committees, we collected the data of 614 patients with stage II-III NSCLC diagnosed in Sichuan Provincial People’s Hospital and Jiangsu Cancer Hospital from January 2015 to December 2018.

Inclusion criteria were as follows: ① patients with primary non-small cell lung cancer in stage II-III according to the 2015 US NCCN Guidelines: Non-Small Cell Lung Cancer (Ettinger et al., 2015); ② patients with good compliance who had at least two follow-up records during the study.

Exclusion criteria were as follows: ① patients with other solid tumors and hematological tumors at the same time; ② patients with a history of thrombosis, severe cardiovascular and cerebrovascular diseases, severe coagulation dysfunction, or severe rheumatic system diseases (including systemic lupus erythematosus); ③ serious complications (including heart failure, respiratory failure, and deep coma caused by non-VTE) or death occurring after enrollment/within 30 days after surgery; ④ patients with uncontrollable neurological, psychiatric, or psychiatric disorders during the follow-up period; ⑤ patients with poor compliance and no follow-up records after the first admission; ⑥ other conditions considered unsuitable for enrollment by the researchers.

### Data Collection

The data collected included paitients’ demographic characteristics, tumor characteristics, laboratory examination reports, records of different interventions received by patients during the study, adverse reactions recorded in medical records, and outcomes (death or survival) collected by a telephone survey in November 2020.

Demographic characteristics included the patients’ age and gender. Tumor characteristics included pathological type and stage of NSCLC; laboratory examination records included hematologic and coagulation parameters. PLT (platelet count) level was the main focus of hematologic examination. Coagulation parameters included FIB (fibrinogen), PT (prothrombin time), APTT (activated partial thromboplastin time), TT (thrombin time interval), and D-Dimer interventions included surgery, chemotherapy, radiotherapy, targeted therapy, immunotherapy, and all kinds of CPM recorded in this study; the outcome was defined as the 2-year survival rate.

### Study Group Assignment and Outcomes

**FIGURE 1:**
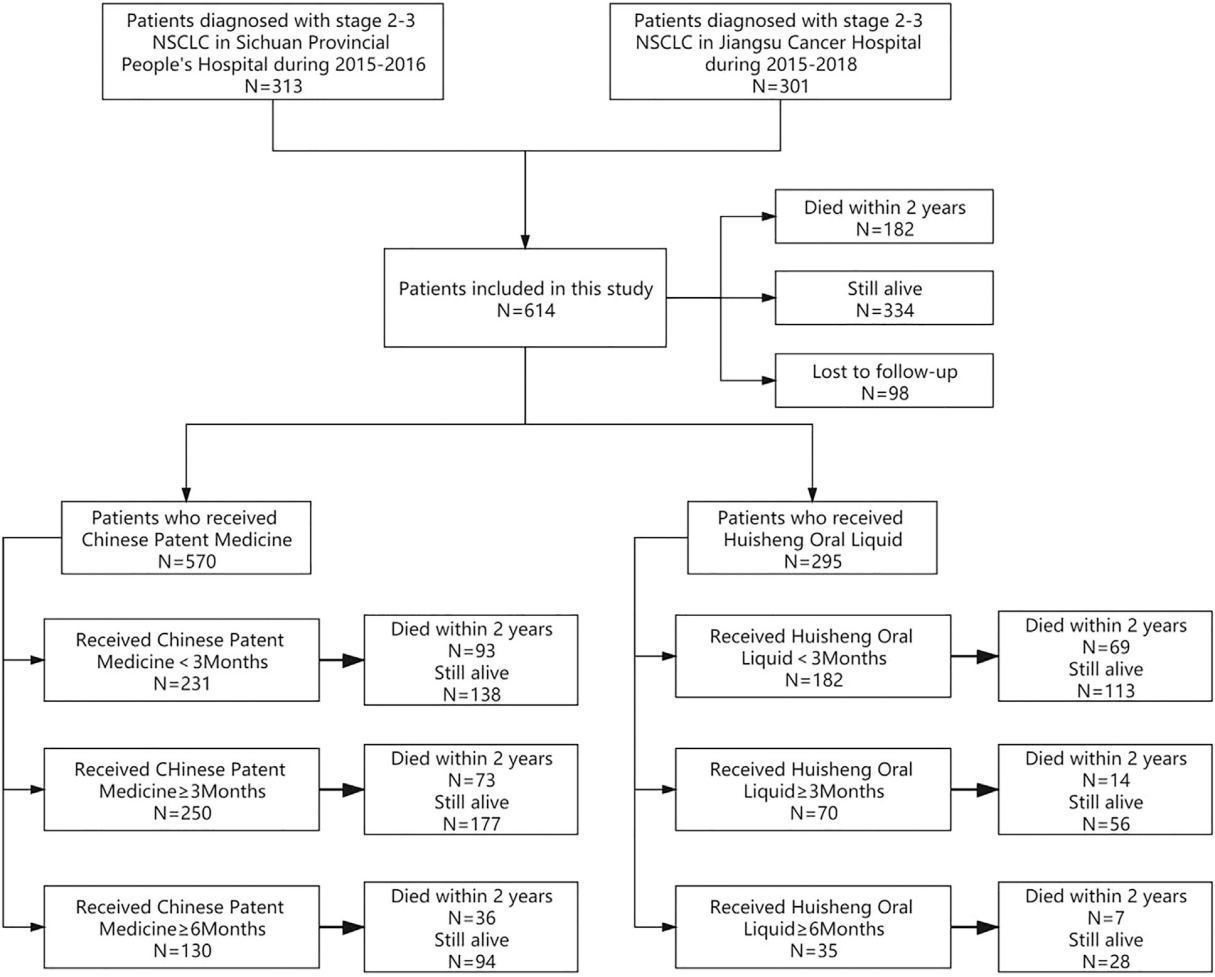
Study group assignment.

According to the different treatment durations of patients who received CPM or HSOL, we divide the data into the following study groups: ① to compare the 2-year survival rate of patients treated with CPM for <3 months and those treated with CPM for ≥3 months and ≥6 months; ② to further compare the 2-year survival rate of patients treated with HSOL for <3 months, ≥3 months, and ≥6 months. The outcome of the study was a 2-year survival rate after diagnosis for each patient. The study group assignment was shown in Figure 1 (Figure 1).

### Statistical Analysis

For the comparison of baseline for quantitative variables between groups, *t*-test and the Wilcoxon rank-sum test with two independent sample comparisons were used. A chi-square test was used for the comparison of baseline of qualitative variables between different groups and the Kaplan–Meier method was used to plot survival curves between different groups. A Log-Rank test was used to compare the 2-year survival rate. A Cochran–Armitage trend test was also used to investigate the relationship between different durations of treatment and outcome in patients treated with HSOL. The Cox proportional-hazards regression model was used to estimate hazard ratios (HRs) with 95% confidence intervals (95% CIs) for each of the independent prognostic factors that may affect the outcome of patients. All of the tests were two-sided, and *p* < 0.05 was considered statistically significant. R 4.0.3 was used for statistical analysis.

## Results

### Overview of All Treatments in All 614 Patients

The treatments patients received are shown in Table 1: out of 614 patients, 390 (63.5%) underwent surgery, 496 (80.8%) received chemotherapy, 121 (19.7%) received radiotherapy, and 78 (12.7%) patients received targeted or immunotherapy. The proportion of patients who had used CPM was the largest, with more than 92.8% of patients receiving CPM and 48.0% of patients treated with HSOL (Table 1).

**TABLE 1:** Overview of all treatment in 614 patients.

Treatment	Number (N)	Frequency (N/614,%)
**Surgery**		
Accepted	390	63.5
Not accepted	224	36.5
**Chemotherapy**		
Accepted	496	80.8
Not accepted	118	19.2
**Radiotherapy**		
Accepted	121	19.7
Not accepted	493	80.3
**Targeted or Immunotherapy**		
Accepted	78	12.7
Not accepted	536	87.3
**Chinese patent medicine**		
Received <3 months	269	43.8
Received ≥3 months	301	49.0
Received ≥6 months	153	24.9
Not received	44	7.2
**Huisheng Oral Liquid**		
Received <3 months	216	35.2
Received ≥3 months	79	12.8
Received ≥6 months	40	6.5
Not received	319	52.0

Treatment frequency = the number of patients treated/number of all patients included in the study (614 patients).

### Comparison of Baseline Data and Survival Analysis of Patients in Different Groups

#### Comparison of Baseline Data Between Patients Treated with CPM for <3 Months and Patients Treated with CPM for ≥3 Months

Among the patients who received CPM, the baselines of the CPM treatment for <3 months group and the CPM treatment for ≥3 months group were comparable. The difference was not statistically significant (*p* > 0.05) (Table 2).

**TABLE 2:** Baseline comparison of CPM use for <3 months and ≥3 months group.

Variable	Treatment duration <3 months	Treatment duration ≥3 months	Statistics	*p* value
**Gender**				
Male	198	243	*χ*^*2*^ = 3.232	*p* = 0.072
Female	69	59		
Age	63.07 (±9.11)	62.87 (±9.03)	*t* = 0.264	*p* = 0.792
**Tumor stage**				
Ⅱ	93	114	*χ*^*2*^ = 0.521	*p* = 0.47
Ⅲ	174	188		
**Pathological types**				
Squamous carcinoma	87	102	Fisher’s Exact Test	*p* = 0.088
Adenocarcinoma	145	179		
Poorly differentiated carcinoma	7	5		
Spindle cell carcinoma	2	0		
Large cell carcinoma	0	1		
Other types	26	15		
**Blood routine**				
PLT	227.55 (±95.13)	238.05 (±91.10)	*t* = *−*1.335	*p* = 0.182
**Coagulation indicators**				
FIB	3.76 (±1.88)	3.63 (±1.12)	*t* = 1.015	*p* = 0.311
D-Dimer	0.89 (±1.74)	0.96 (±2.10)	*t* = *−*0.303	*p* = 0.762
TT	18.45 (±2.63)	18.14 (±2.77)	*t* = 1.544	*p* = 0.123
PT	12.10 (±2.12)	12.57 (±2.85)	*t* = *−*1.91	*p* = 0.06
APTT	27.10 (±4.12)	26.95 (±5.27)	*t* = 0.325	*p* = 0.745

t indicated the *t* value, and X^2^ indicated the chi-square value. *p* < 0.05, there was a statistical significance between the two groups.

#### Survival Analysis of Patients Treated with CPM for <3 Months and Patients Treated with CPM for ≥3 Months

**FIGURE 2:**
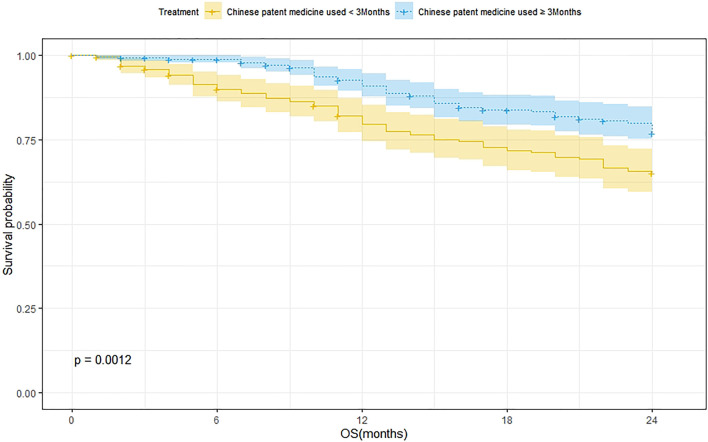
Survival analysis of patients treated with CPM for <3 months and patients treated with CPM for ≥3 months.

According to the treatment duration of CPM, a total of 239 patients were treated for <3 months and 75 of them died. The 1-year and 2-year survival rates were 79.8% and 65.2%, respectively. The median survival was not reached. As for the 289 patients treated with CPM for ≥3 months, 60 of them died, and the 1-year and 2-year survival rates were 91.1% and 77.1%, respectively. The median survival was not reached. The Kaplan–Meier was used to analyze the data of two groups, and the result showed that there was a statistically significant difference between the two groups (*p* = 0.0012). The 2-year survival rate of patients treated with CPM for ≥3 months was higher than that of patients treated with CPM for <3 months (Figure 2).

#### Comparison of Baseline Data Between Patients Treated with CPM for <3 Months and Patients Treated with CPM for ≥6 Months

Among the patients who received CPM, the baselines of the patients treated with CPM for <3 months group and the patients treated with CPM for ≥6 months group were comparable. The difference was not statistically significant (*p* > 0.05) (Table 3).

**TABLE 3:** Baseline comparison of CPM use for <3 months and ≥ 6 months group.

Variable	Treatment duration <3 months	Treatment duration ≥6 months	Statistics	*p* value
**Gender**				
Male	198	121	*χ*^*2*^ = 1.293	*p* = 0.255
Female	69	32		
Age	63.07 (±9.11)	63.74 (±9.30)	*t* = *−*0.713	*p* = 0.476
**Tumor stage**				
Ⅱ	93	56	*χ*^*2*^ = 0.133	*p* = 0.715
Ⅲ	174	97		
**Pathological type**				
Squamous carcinoma	134	47	Fisher’s Exact Test	*p* = 0.630
Adenocarcinoma	241	96		
Poorly differentiated carcinoma	11	3		
Spindle cell carcinoma	1	0		
Large cell carcinoma	0	0		
Other types	33	7		
**Blood routine**				
PLT	227.55 (±95.13)	233.16 (±95.18)	*t* = *−*0.577	*p* = 0.564
**Coagulation indicators**				
FIB	3.76 (±1.88)	3.61 (±1.17)	*t* = 0.860	*p* = 0.390
D-dimer	0.89 (±1.74)	1.28 (±2.85)	*t* = *−*1.224	*p* = 0.222
TT	18.45 (±2.63)	18.16 (±2.61)	*t* = 1.051	*p* = 0.294
PT	12.10 (±2.12)	12.45 (±2.81)	*t* = *−*1.258	*p* = 0.209
APTT	27.10 (±4.12)	26.74 (±5.49)	*t* = 0.657	*p* = 0.512

#### Survival Analysis of Patients Treated with CPM for <3 Months and Patients Treated with CPM for ≥6 Months

**FIGURE 3:**
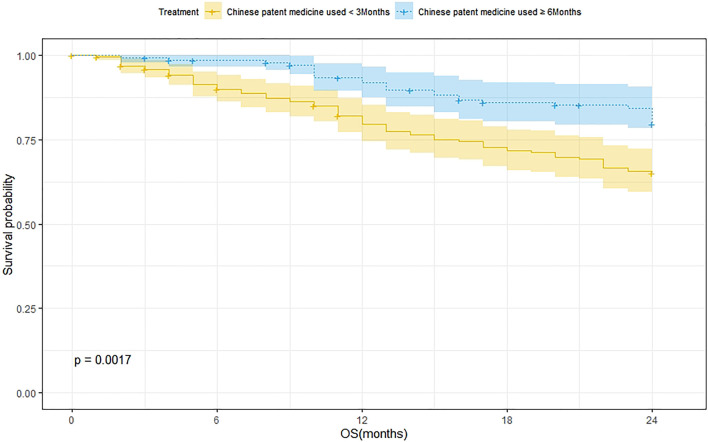
Survival analysis of patients treated with CPM for <3 months and patients treated with CPM for ≥6 months.

A total of 239 patients were treated with CPM for <3 months and 75 of them died. The 1-year and 2-year survival rates were 79.8% and 65.2%, respectively. The median survival was not reached. As for the 144 patients treated with CPM for ≥3 months, 27 of them died. The 1-year and 2-year survival rates were 92.0% and 79.7%, respectively. The median survival was not reached. The Kaplan–Meier method was used to analyze the data of the two groups. The result showed that there was a significant difference between the two groups (*p* = 0.0017). The 2-year survival rate of patients treated with CPM for ≥3 months was higher than that of patients treated with CPM for <3 months (Figure 3).

#### Comparison of Baseline Data of Patients Treated with HSOL for <3 Months and ≥3 Months

Among the patients who received HSOL, the baselines of the patients treated with HSOL for <3 months group and the patients treated with HSOL for ≥6 months group were comparable. The difference was not statistically significant (*p* > 0.05) (Table 4).

**TABLE 4:** Baseline comparison between the HSOL use for <3 months and ≥3 months group.

Variable	Treatment duration <3 months	Treatment duration ≥3 months	Statistics	*p* value
**Gender**				
Male	170	57	*χ*^*2*^ = 1.40	*p* = 0.237
Female	46	22		
Age	62.58 (±9.22)	62.57 (±9.99)	*t* = 0.01	*p* = 0.99
****Tumor stage****				
Ⅱ	87	30	*χ*^*2*^ = 0.128	*p* = 0.720
Ⅲ	129	49		
**Pathological type**				
Squamous carcinoma	81	20	Fisher’s Exact Test	*p* = 0.199
Adenocarcinoma	116	52		
Poorly differentiated carcinoma	3	3		
Spindle cell carcinoma	1	0		
Large cell carcinoma	1	0		
Other types	14	4		
**Blood routine**				
PLT	231.65 (±87.60)	215.53 (±94.88)	*t* = 1.358	*p* = 0.176
**Coagulation indicators**				
FIB	3.67 (±1.23)	3.57 (±1.19)	*t* = 0.583	*p* = 0.56
D-dimer	0.71 (±1.22)	1.17 (±4.01)	*t* = *−*1.02	*p* = 0.311
TT	18.25 (±2.77)	17.57 (±2.81)	*t* = 1.805	*p* = 0.07
PT	12.53 (±2.77)	12.70 (±3.28)	*t* = *−*0.366	*p* = 0.714
APTT	26.87 (±4.98)	27.69 (±4.61)	*t* = *−*1.074	*p* = 0.284

#### Survival Analysis of Patients Treated with HSOL for <3 Months and ≥3 Months

**FIGURE 4:**
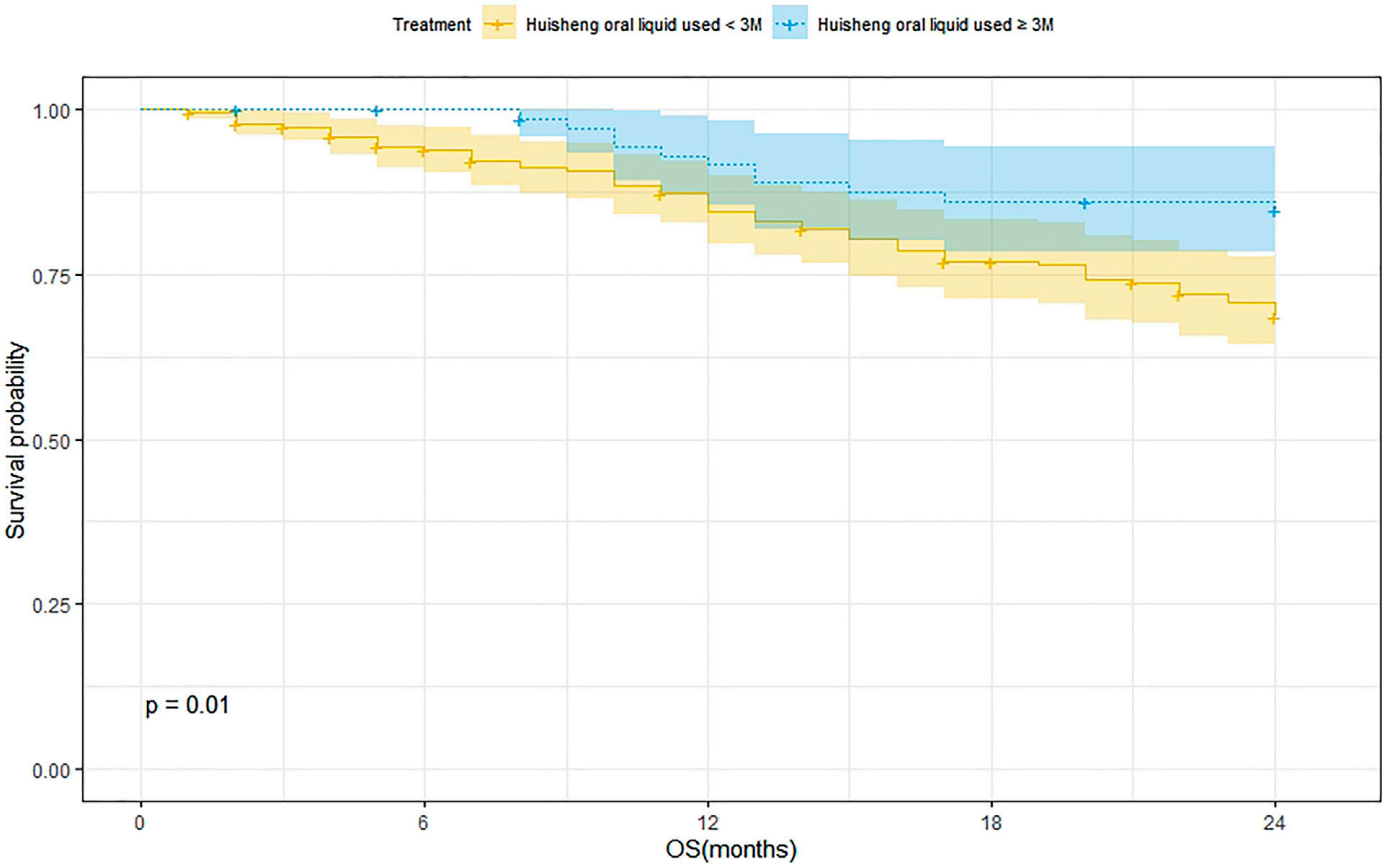
Survival analysis of patients treated with HSOL for <3 months and HSOL for ≥3 months.

A total of 202 patients were treated with HSOL for <3 months and 58 of them died. Moreover, the 1-year and 2-year survival rates were 84.7% and 68.5%, respectively. The median survival was not reached. As for the 76 patients who received HSOL for ≥3 months, 11 of them died. The 1-year and 2-year survival rates were 91.7% and 84.7%, respectively. The median survival was not reached. The Kaplan–Meier method was used to analyze the data of the two groups. The result has demonstrated that there was a statistically significant difference in the 2-year survival rate between the patients treated with HSOL for <3 months and ≥3 months (*p* = 0.01). It can be concluded that the 2-year survival rate of patients who received HSOL for ≥3 months is higher than that of patients who received HSOL for <3 months (Figure 4).

#### Comparison of Baseline Data of Patients Treated with HSOL for <3 Months and HSOL for ≥6 Months

There was a significant difference in the number of patients treated with HSOL for <3 months and ≥6 months. Except for gender, the baselines of the two groups were comparable, with no statistical significance (*p* > 0.05) (Table 5).

**TABLE 5:** Baseline comparison between the HSOL use for <3 months and ≥6 months group.

Variable	Treatment duration <3 M	Treatment duration ≥6 M	Statistics	*p* value
**Gender**				
Male	170	25	*χ* ^*2*^ *= 4.880*	*p = 0.027*
Female	46	15		
Age	62.58 (±9.22)	62.80 (±8.78)	*t = −1.407*	*p = 0.161*
**Stage**				
Ⅱ	87	16	*χ* ^*2*^ *= 0.001*	*p = 0.974*
Ⅲ	129	24		
**Pathological types**				
Squamous carcinoma	81	11	Fisher’s Exact Test	*p = 0.672*
Adenocarcinoma	116	26		
Poorly differentiated carcinoma	3	0		
Spindle cell carcinoma	1	0		
Large cell carcinoma	1	0		
Other types	14	3		
**Blood routine**				
PLT	231.65 (±87.60)	207.33 (±93.73)	*t = 1.592*	*p = 0.113*
**Coagulation index**				
FIB	3.67 (±1.23)	3.67 (±1.23)	*t = −0.032*	*p = 0.975*
D-dimer	0.71 (±1.22)	0.46 (±0.55)	*t = 0.728*	*p = 0.468*
TT	18.25 (±2.77)	18.02 (±1.90)	*t = 0.493*	*p = 0.622*
PT	12.53 (±2.77)	11.56 (±1.91)	*t = 1.752*	*p = 0.081*
APTT	26.87 (±4.98)	28.45 (±4.54)	*t = −1.545*	*p = 0.124*

#### Survival Analysis of Patients Treated with HSOL for <3 Months and HSOL for ≥6 Months

**FIGURE 5:**
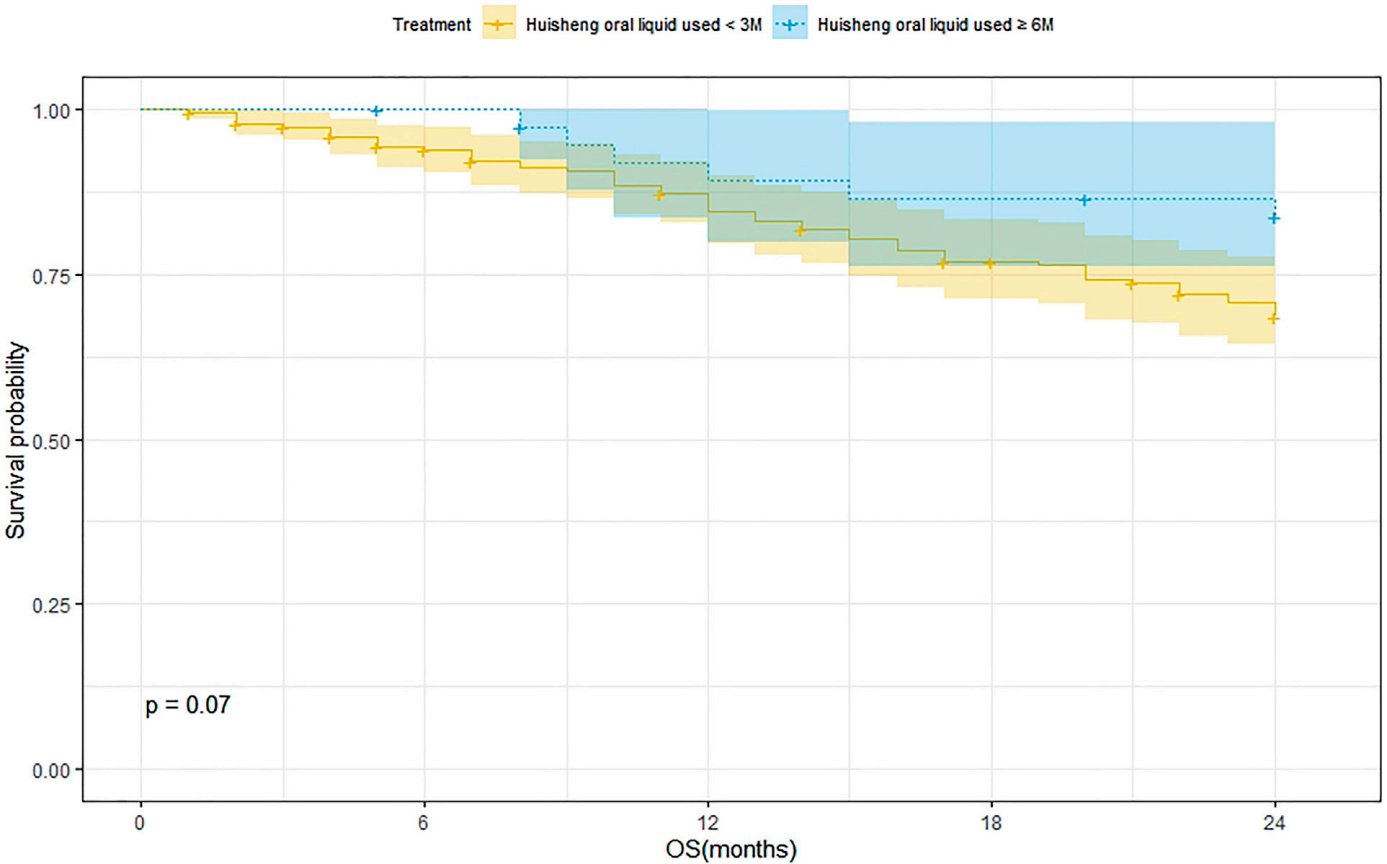
Survival analysis of patients treated with HSOL for <3 months and HSOL for ≥6 months.

A total of 202 patients were treated with HSOL for <3 months, and 58 of them died. The 1-year and 2-year survival rates were 84.7% and 68.5%, respectively. The median survival was not reached. A total of 39 patients were treated with HSOL for ≥6 months, and six of them died. The 1-year and 2-year survival rates were 89.3% and 83.8%, respectively. The median survival was not reached. The Kaplan–Meier method was used to analyze the data of the two groups. The 2-year survival rate of the patients treated with HSOL for ≥6 months was superior to that of the patients treated with HSOL for <3 months. However, the total number of patients and the number of patients with outcomes occurring in HSOL treated for ≥6 months was too, small, resulting in the low power of the test (1-β = 0.33). Therefore, there was no significant difference between the two groups (*p* = 0.07) (Figure 5).

### Trend Analysis for Patients Treated With HSOL for Different Durations

Of all 614 patients in this study, 295 patients used HSOL during this study. The patients were grouped into HSOL treatment for <3 months, 3 months ≤ HSOL treatment duration <6 months, and HSOL treatment for ≥6 months. The 2-year survival rate of each group was 68.1%, 82.1, and 82.5%, respectively. The Cochran–Armitage trend test revealed that the increasing trend of the 2-year survival rate was statistically significant (*p* = 0.01). The 2-year survival rate of patients increased with the treatment duration of HSOL (Table 6).

**TABLE 6:** Trend of the 2-year survival rate of patients treated with HSOL for different durations.

Duration (months)	Dead (n, %)	Alive (n, %)	Total (n, %)
Huisheng <3 M	69 (31.9%)	147 (68.1%)	216 (73.2%)
3 M ≤ Huisheng <6 M	7 (17.9%)	32 (82.1%)	39 (13.2%)
Huisheng ≥6 M	7 (17.5%)	33 (82.5%)	40 (13.6%)
Total	83 (28.1%)	212 (71.9%)	295 (100%)

### Cox Proportional-Hazards Regression Model

Cox regression showed that in all 614 patients, surgery (HR = 0.48, 95% CI = [0.34, 0.68]), chemotherapy (HR = 0.46, 95% CI = [0.31, 0.67]), and HSOL treatment for ≥3 months were protective factors (HR = 0.48, 95% CI = [0.27, 0.88]), whereas patient’s gender being male (HR = 1.59, 95% CI = [1.01, 2.50]) and FIB ≥4 g/L (HR = 1.95, 95% CI = [1.37, 2.77]) were risk factors. The tumor stage was generally considered a risk factor for the outcome, but we did not validate this theory in the current study due to the limitations of retrospective data and possible collinearity among the variables. In addition, surgery, chemotherapy, targeted therapy, and immunotherapy were considered essential factors affecting the prognosis of the patients. Due to the complexity of the interventions that patients received in real-world conditions, patients may receive chemotherapy, targeted or immunotherapy after surgery, which may affect the assessment of HSOL efficacy to a certain extent. However, all included patients were treated in accordance with NCCN guidelines, and the Cox regression model was also used to adjust for interference with these treatments. Finally, D-Dimer was considered a covariate that should be included in this model, but we did not include it due to the excessive data missing (more than 50%) (Table 7).

**TABLE 7:** Multi-variable Cox regression model.

Variable	Hazard ratio (95% CI)	*p* value
**Gender**		
Male	1.00 (reference)	*p* = 0.048^a^
Female	1.59 (1.01–2.50)	
Age	1.00 (0.98–1.02)	*p* = 0.974
**Tumor stage**		
Ⅱ	1.00 (reference)	*p* = 0.21
Ⅲ	1.26 (0.87–1.83)	
**PLT**		
PLT<300	1.00 (reference)	*p* = 0.92
PLT≥300	0.98 (0.63–1.52)	
**FIB**		
FIB<4 g/L	1.00 (reference)	*p* < 0.001^a^
FIB≥4 g/L	1.95 (1.37–2.77)	
**TT**		
TT < 16 s	1.00 (reference)	*p* = 0.13
TT ≥ 16 s	0.72 (0.47–1.10)	
**Surgery**		
Not accepted	1.00 (reference)	*p* < 0.001^a^
Accepted	0.48 (0.34–0.68)	
**Chemotherapy**		
Not accepted	1.00 (reference)	*p* < 0.001^a^
Accepted	0.46 (0.31–0.67)	
**Targeted or immunotherapy**		
Not accepted	1.00 (reference)	*p* = 0.24
Accepted	0.73 (0.43–1.24)	
**Huisheng Oral Liquid**		
Received < 3 months	1.00 (reference)	*p* = 0.01^a^
Received ≥ 3 months	0.48 (0.27–0.88)	

a There was a statistical significance between the two groups.

### Safety Assessments

No adverse reactions related to HSOL were found during the study. All adverse reactions recorded in the HIS (Hospital Information System) were physician-judged adverse reactions related to radiotherapy and chemotherapy during the course of treatment. The Chinese National Adverse Drug Reaction Information and Pharmacovigilance notice related to Hisheng Oral Liquid was not received and no serious adverse reactions have been reported in current clinical literature.

## Discussion

It took a long time for traditional Chinese medicine to exert its efficacy in diagnosis and treatment, and the short-term treatment did not objectively reflect the difference and variability of individual patients under the trend of overall treatment efficacy, which also makes it difficult to uncover any hidden diagnostic and therapeutic patterns of CPM (Shi et al., 2020). The concept of taking a “long-term” perspective in the evaluation of treatment effect of CPM coincides with the results found earlier by Lin and Yang (Xu et al., 2017; Wang et al., 2019) on the improvement of survival outcomes of patients with non-small cell lung cancer and colon cancer after long-term treatment with CPM. In addition, Chen et al. (2019) have found that the onset of tumors disrupted the coagulation-fibrinolysis balance under normal conditions, resulting in hypercoagulability and the generation of thrombin. Thrombin catalyzed the conversion of FIB into insoluble fibrin, which provided structural support and facilitated the growth, infiltration, and metastasis of tumor cells. Therefore, based on the “long-term” perspective of CPM treatment and the possible mechanism of tumor progression, this study explored the effect of different treatment durations of CPM and HSOL on the 2-year survival rate of NSCLC patients by a retrospective study. We found that CPM treatment for ≥3 months was associated with an improved 2-year survival rate in NSCLC patients. In further exploration of CPM, HSOL used for ≥3 months also showed an improvement in 2-year survival rate in NSCLC patients. Both results were better than that of those patients with stage II-III NSCLC who were observed only after surgery or treated with chemotherapy or radiotherapy alone (Winton et al., 2005; Akamatsu et al., 2014). In addition, we graded and grouped the patients treated with HSOL according to their treatment durations. The result of the Cochran–Armitage trend test showed that the 2-year survival rate increased while the treatment duration of HSOL increased. We also hoped to observe the effect of CPM on PFS (progression-free survival), but this result has not been achieved due to incomplete data and the difficulty in defining time and first recurrence or metastasis of patients in HIS. Nonetheless, this retrospective study provided preliminary evidence to evaluate the relationship between different treatment durations of CPM and the survival outcomes of patients and explore the factors affecting the prognosis of patients.

Due to the limitations in this study, our study provided very limited data support for the impact of CPM treatment on survival outcomes of patients with stage II-III NSCLC. First, as a retrospective study, we collected data from HIS, but due to the complexity of each individual treatment and missing data in the medical records, it was difficult to restore 100% of the actual treatment of patients in the past, and we could only use these data as much as possible to obtain a relatively reliable result. Second, some unmeasured covariates, such as education, income, smoking, and bodily performance status, might also affect the study results, but for the covariates that have been collected, we have already adjusted their impact on the results by establishing a Cox proportional-hazards regression model. Finally, it was also difficult to show the effect of HSOL alone due to the various treatments received by patients during the period of the study. Thus, we are now planning to conduct a more rigorous prospective study to determine the effect of HSOL on the survival outcomes of patients with stage II-III NSCLC.

## Data Availability

The raw data supporting the conclusion of this article will be made available by the authors without undue reservation.
